# Cutting Techniques in the Fish Industry: A Critical Review

**DOI:** 10.3390/foods11203206

**Published:** 2022-10-14

**Authors:** Wenbo Liu, Jiaqi Lyu, Di Wu, Yupeng Cao, Qingquan Ma, Yuzhen Lu, Xin Zhang

**Affiliations:** 1 Coastal Research & Extension Center & Department of Agricultural and Biological Engineering, Mississippi State University, Pascagoula, MS 39567, USA; 2 Department of Mechanical Engineering, Stevens Institute of Technology, Hoboken, NJ 07030, USA; 3 Department of Chemical Engineering and Materials Science, Stevens Institute of Technology, Hoboken, NJ 07030, USA; 4 Department of Electrical and Computer Engineering, Stevens Institute of Technology, Hoboken, NJ 07030, USA; 5 Department of Civil and Environmental Engineering, New Jersey Institute of Technology, Newark, NJ 07102, USA; 6 Department of Agricultural and Biological Engineering, Mississippi State University, Starkville, MS 39762, USA; 7 Department of Biosystems and Agricultural Engineering, Michigan State University, East Lansing, MI 48824, USA

**Keywords:** fish cutting, fish processing, waterjet cutting, machine vision, artificial intelligence

## Abstract

Fish and fishery products are among the most important sources of nutritional components for human health, including high-quality proteins, essential vitamins, minerals, and healthy polyunsaturated fatty acids. Fish farming and processing technologies are continuously evolving to improve and enhance the appearance, yield, and quality of fish and fish products from farm to fork throughout the fish supply chain, including growth, postharvest, treatment, storage, transportation, and distribution. Processing of fish involves a period of food withdrawal, collection and transportation, the process of stunning, bleeding, chilling, cutting, packaging, and byproduct recycling. Cutting is a set of crucial operations in fish processing to divide the whole fish into smaller pieces for producing fish products (e.g., fish fillets, steaks, etc.). Various techniques and machinery have been introduced in the field to advance and automate cutting operations. This review aims to provide a comprehensive review of fish cutting techniques, machine vision and artificial intelligence applications, and future directions in fish industries. This paper is expected to stimulate research on enhancing fish cutting yield, product diversity, safety and quality, as well as providing advanced solutions for engineering problems encountered in the fish industry.

## 1. Introduction

Fish production is a multibillion-dollar industry worldwide since seafood is one of the major food recourses which create billions of dollars of value per year. Studies have been carried out to discuss the fishery and the related techniques in different countries [1,2,3]. According to the latest data from Food and Agriculture Organization, total world fisheries and aquaculture production reached 177.8 million tons in 2020, with an increase of 9.3% compared with 2010, and at an estimated sale value of USD 406 billion [4]. Due to the high prices of fish products and the varying needs of the consumer, it is critical to carry out the fish-processing tasks such that the end product is of high quality. Fish processing is the process that turns the raw fresh fish into fish products we can buy in the market or use in manufacturing other fish-related products [5]. The typical fish processing begins right after the capture of fish. It involves fish receiving (capturing), stunning, bleeding & chilling, grading, deheading (beheading), scaling, filleting, skinning, trimming & portioning, mincing, byproduct recycling, and packaging [6,7,8]. Briefly, once the fish is captured, the sorting of different species will be conducted. Then, the fish is transferred to the nearest processing facilities, followed by stunning and grading according to the size and quality of the fish. The stunned fish is bled and washed off by clean circulating water. After bleeding, the fish is chilled and then gutted. The bleeding and chilling process can lower bacteria and enzyme activity to extend shelf-life and quality [9,10]. The gutting process will remove the inter organs and clean the body cavity [11]. Then the fish is further scaled, beheaded, filleted, and skinned to produce fish fillets. The fish fillets need to be trimmed, portioned, sliced into pieces, or minced, depending on the requirement of the final products. The byproduct during the gutting and cutting steps will be collected for other use [12]. Among these processing works, cutting steps play major roles since they involve deheading, filleting, trimming, skinning, and portioning. Hence, the study and discussion of the cutting methods are important for the development of the fish industry.

Fish processing makes the major waste of fish come from the “butchering of fish,” during which the fish are gutted and cut into fish products. This process involves the removal of non-edible portions like viscera, head, and tail that would cause up to 70% loss of fish, which is partially caused by inefficient cutting operations. In traditional fish cutting, humans played a significant role since the cutting process was completed via laborers using knives [13]. Like other food industries, fish cutting used to be labor-intensive. However, fishery operation is seasonal, which leads to difficulty in maintaining experienced laborers. Moreover, the routine and stinking environment of fish cutting somehow discourage the workers’ morale. Therefore, it is desirable to increase the application of automation in the fish-cutting process for higher productivity and economic benefit [14]. The most commonly employed method for cutting fish is to use metal blades [13,15,16,17]. In the past three decades, research about automatic fish cutting mainly focused on how to preciously (1) detect the size of the fish [18], (2) position the fish head [19], (3) decrease the waste of raw fish [20,21], and (4) reduce the error rate of the automatic system [22]. Metal blade cutting is indeed effective in cutting operations and cost-efficient in terms of equipment maintenance. However, the force applied for cutting reduces the quality of sections. The considerable amount of cutting debris also leads to a large waste of raw fish [23]. Efforts have been invested in investigating and optimizing the cutting mechanism [24], cutting performance, processing speed, motor power, and processable fish species [25]. However, the potential heavy metal food contamination is expected under the existence of an aqueous medium [26].

Novel techniques have been invented to overcome the drawbacks of metal blade cutting. Water-jet cutting is a well-developed cutting technique which frequently applied in various industries. Recently, it has been largely applied for food cutting, including fish [27]. The rheological properties of fish meat are significantly different from common workpieces such as metal, rock, or wood [28]. With the aid of developing automatic robotic arms, waterjet cutting is believed to be faster and able to handle fish meat with bone, skin, and fiber together with fewer bone shatters and bleeding [29,30]. Compared with knife cutting, waterjet cutting exhibited advantages in cutting efficiency and quality for food processing [31]. This method mainly utilizes the high pressure of the waterjet coming through a die with a certain shape to separate the workpieces [32]. To enhance the cutting performance, certain abrasives are added to the water [33,34]. The modern waterjet cutting machine can cut intricate shapes with high precision [24]. It has the capability to operate at low temperatures, which is very important for fish cutting [35]. It is believed that waterjet cutting will become one of the major cutting methods in the food industry [8]. To further reduce the reliance on manual work, the machine vision technique has been widely utilized in modern fish automation processing systems. The machine vision technique implements optical sensors to monitor the status of fish processing. In most instances, the images of the fish’s body should be acquired, stored, and analyzed. The useful information would be extracted by the machine vision algorithm for fish processing lines, like species, fish size, and body shape [36]. In the fish-cutting processes, such as de-heading and filleting, facilities require precise identification and segmentation of fish bodies. Inspired by the outstanding image process performances of artificial intelligence (AI), modern fish automation processing systems are gradually employing AI algorithms to identify the patterns of fish bodies and guide the subsequent fish-cutting processes [37].

In this review article, we considered these focused questions: what kind of advanced technologies are used at present and being considered for fish-cutting operations? How to improve the fish-cutting yield, quality, and efficiency? How to realize fish-cutting automation? Initially, several major scientific databases, including Web of Science, IEEE Xplore, ScienceDirect, Google Scholar, and Springer, were searched to collate the literature. Then we deselected some databases with limited and repeated articles since the databases with broader coverage may cover the articles coming from the other databases with limited and repeated articles. Finally, this review considered three mainstream scientific databases (IEEE Xplore, ScienceDirect, and Google Scholar) and one open-access database (arXiv). For the search strategy and inclusion criteria, we first searched the databases with individual keywords such as “waterjet fish cutting,” “automatic fish cutting,” “robotics for fish deheading,” “automatic fish trimming,” “fish cutting machine design,” “machine vision in fish cutting,” and “artificial intelligence in fish cutting.” Then similar keywords were grouped with AND as search string combinations, like “fish-cutting yield optimization” AND “fish-cutting quality assessment,” and “image processing in fish-cutting” AND “computer vision applications of fish processing.” The keywords in diverse fields were also grouped with OR, such as “automation technologies for fish processing,” OR “robotic trimming of fish fillets,” and “deheading cuts used in fish processing” OR “fish-skinning machine design.”

Although there are some review papers focusing on advanced cutting techniques for solid food [38] and fish processing automation [8], there is no paper, especially reviewing the cutting techniques for fish. The uniqueness of this paper is focusing on fish cutting steps (deheading, filleting, skinning, portioning, and trimming) and their corresponding cutting mechanism and techniques, blade design, and performance effect factors. Other related advanced technologies (waterjet cutting, machine vision and AI) are also presented with their applications in fish cutting. Finally, fish cutting machines and technologies and future perspectives are described and summarized.

## 2. Modern Fish Cutting

### 2.1. Metal Blade Cutting Machines

**Figure 1:**
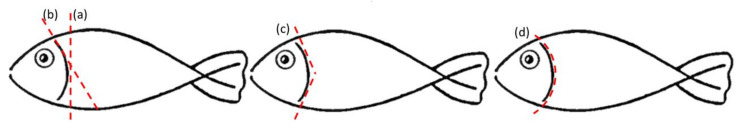
Schematic illustration of fish de-heading position: (**a**) Straight cutting, (**b**) Slant cutting, (**c**) V-cutting, and (**d**) Round cutting along the gill area.

Metal blade cutting is the most widely used cutting method in the fish industry. In the fish processing line, the deheading of fish is usually the first cut operation. The purpose of this operation is to separate the high-value fillets from the relatively low-value head. To maximize the commercial values, the deheading machines should leave the maximum amount of meat on the fillet and ensure no part of the fish gills, and the head skeleton is included in the fillet [39,40,41,42]. The main deheading position can be classified into four classes, as shown in Figure 1, namely straight cutting, slant cutting, V-cutting, and round cutting along the gill area [43,44]. Commercial machines, like Baader 166, have been used in industry and research [39]. Buckingham et al. [40] designed a robotic solution for fish deheading called Robofish 2. The V-cutting was performed in this machine. To perform the optimum cutting, the shape of the fish changed during the deheading process. The fish was in a back-down position when it arrived. Then the head was gripped and bent to allow the metal blades to cut the flesh. An in-line weighing component was built-in this deheading machine to perform a unique cutting for each fish. Therefore, the Robofish 2 could have high cutting reliability. Ketels [41] designed a machine that could align the fish in a row in the same direction. A cutting blade driven by a motor can perform straight or slant cutting in the desired direction with the help of a designed pressure part. Rodríguez and Martínez [45] developed a method to perform round cutting for the fish silhouette. The round cutting can follow the operculum’s edge to acquire the lowest meat loss. Similar to large fish cutting, deheading is the initial cutting process for small-sized fish, like sardine, horse mackerel and mackerel. However, due to the large size deviation, the deheading machines for small-sized fish need to be redesigned to increase production efficiency. The SEAC FPM-200, which is mainly designed for cutting small-sized fish for canning, aligns several small-sized fish and performs deheading simultaneously [46]. PN-200 of Pisces Fish Machinery Inc. is designed to remove head and viscera for small fish like Sardine, Anchovy, Smelt and others under 200 mm in length.

**Figure 2:**
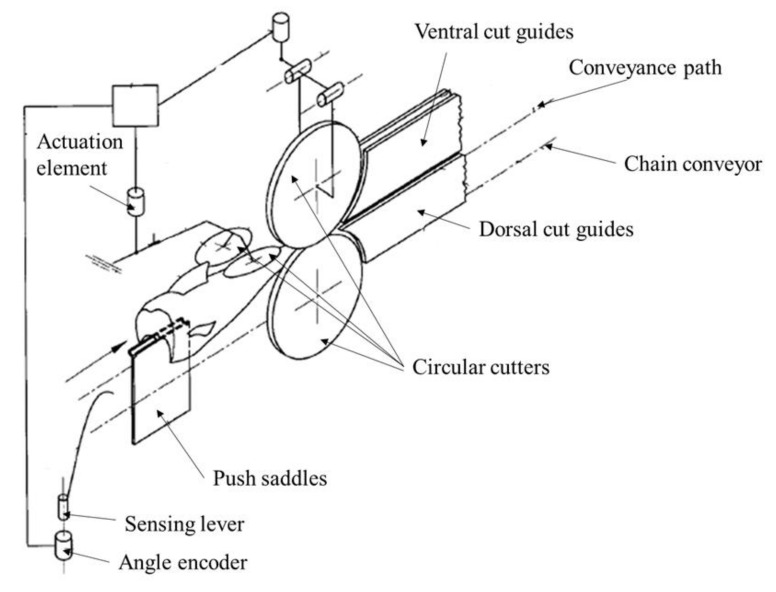
The representation of filleting machine for fish of Siluridae species (Braeger and Scherch, 2001).

For the filleting operation, as the majority of fish is symmetrical, each procedure is conducted with a pair of symmetrical knives. Modern filleting machines are fast, customized to individual fish species, and simple to adjust for various fish sizes. Currently, some machines for producing high-yielding fillets have the capability to handle short-bodied fish species, including salmon, tilapia, trout, croaker, arctic char, bar ramundi, snappers, walleye pike, and striped bass [47,48]. Therefore, these machines could achieve higher yields compared to traditional human-based filleting. The design of automatic machines is flexible. However, if the shape or size of feeding fish is out of the designed working range of filleting machines, it could result in reduced yield and even damage the fish and waste the fillets. During the filleting process, the backbone and fins are removed normally. Based on the product’s specifications, if all the intermuscular bones are required to be removed, it is known as V-cut fillets, which could result in lowering yield (as much as 25%) [49]. Different machines were designed using metal blades to perform fish fillet cutting [50,51,52,53,54]. The main concept is to fix the fish without head and guts in a stable position. Then the blades are implemented to cut the meat from the frame bone. To minimize the meat waste during the filleting process, several blades would be designed into the system, and each of them is configured to cut a distinct segment. For example, a filleting system is designed, as shown in Figure 2 [50]. An endlessly rotating transport conveyor with saddle-shaped support bodies was designed to receive the fish and convey them along the transport direction [55]. In this design, the first two circular blades (tools 17 and 18) were designed to remove the dorsal fin. Then based on the bone structure of the fish, the angle of the following four blades (tools 9–11) could be adjusted to cut fillet meat. Specialized methods and systems designed for processing some common fish species, such as salmon, integrate and enable deheading, filleting, and gutting in a particularly reliable and efficient manner [56,57,58]. Besides, fillet gapping is a significant problem for the filleting process. It is different for fillets with gapping to be processed and sold. Although the causes of fillet gapping are not well understood, some actions could reduce the possibility of gapping, like rapid cooling, harvesting fish in a rest state, minimal harvest handling, season, rapid expedition to the market, etc. [49,59,60]. Production yield is a metric to evaluate the performance and effectiveness of an operation in the production lines. The fillet yield depends on the species and the structural anatomy. Fish with large heads and frames compared to the musculature would have a lower yield than the ones with smaller heads and frames. The well-chilled fish can result in a higher yield than fish at room temperature. The fish filleting machine, PASFF-110, manufactured by Peruza, is designed for small-sized fish, such as sprats, anchovies, capelin and other fish in 9–14 cm [7,61]. It could make “butterfly” type or single fillets or cut off the belly as needed. The TOYO-167 from TOYO SUISAN KIKAI is designed for fillet-cutting small-sized fish, like sardine, horse mackerel, and mackerel pike.

Fish skinning machines are equipped with metal blades as well. Traditionally, the de-skinning processes could be conducted by gas flame (substation of NaOH and HCl) or steam [62]. In recent years, the mechanism of most fish skinners would press the fillets on the blades to remove the skin from the fillets [63]. A typical fish skinner consists of a feeding device, a dispatching device, a rotationally-driven separation device with a driver roller, and a main blade for removing the fish skin. The cutting gap between the main blade and the driver roller can be controlled by detecting fish fillet size [64,65]. To get a better quality of the fillets as value-added products, the deep-skinning process can remove the skin with a layer of subcutaneous fat [66,67]. Due to the relatively simple structure, some fish skinning machines are small and can be placed directly on the processing table. The well-known fish skinning machines are mainly from Baader, Cretel, Nock, and Trio [68]. Joensen and Olsen [69] compared the skinning machine Baader 51 and Trio FDS 105. The differences between these two skinning principles are the fillet feeding direction and skinning temperature. The fillet should be fed into the Baader 51 with the skin side down and Trio FDS 105 with the skin side up. In the Trio FDS 105, the skin would be frozen to a cold drum and then cut by the rotating blades. The Baader 51, like most skinners, used a drum to press the fillet to the blades. The results indicate that the fillet gapping increased after using FDS 105 compared with Baader 51. Arnþórsdóttir et al. [70] compared Baader 51 and Skaginn-skinner S3 machines. The super-chilled fillets using Skaginn S3 with Combined Blast and Contact (CBC) technique could have fewer gapping issues and fresher appearances. However, the fish skinning machines are also shown potential damage. Waterston and Holmes [71] studied the hand trauma caused by fish skinning machines. For small-sized fish, the ST600V and ST700V from STEEN can be implemented to remove small-sized fish skin.

To satisfy the specifications of a fish product, the fillets are trimmed and portioned. The trimming operations are aimed at removing the defects and unwanted regions of fish (bones, fin, belly fat area, etc.) and correcting the shape of the fillet [7]. In the automatic fish processing lines, machines are designed for trimming operations. Baader 988, a well-known high-speed auto-trimming machine in the industry, was studied by Ørnholt-Johansson et al. [72]. Based on the requirements, the trimming machine implements rotatory blades to cut the fillets according to pre-set demands. They found that the Baader 988 could achieve 88.0% ± 9.3% weight of fed salmon fillets. After that, fillets can be further portioned to create various products. Because the different parts of a fillet represent the different qualities of meat, the portioning operation is necessary for commercial benefit. The loin is thought to be the most valuable part of the fillet. Usually, the belly flap would fetch the lowest price. Commonly, the cod fillets are portioned in loins, centercuts, belly flaps, and tails. Mathiassen et al. [73] introduced machines in automatic fish processing lines. The Marel I-Cut line of products are metal knife-based portioning machines. The control system of them could change the cutting angle and speed based on the weight or dimensions of feeding non-frozen fillets. Slicing fish fillets is also included in some industrial production lines [7]. Thicker fish fillets can be further sliced with a horizontal slicing motion [74,75]. Whole fish can be vertically sliced or cut to produce fish steak with bone inside [76,77]. The measurements and estimation of weight and volume have been applied as a solution for slicing portions of whole fish and fish fillets [78,79]. Advanced slicer for producing a wide range of salmon products at high speed is designed to provide flexible multi-angle slicing motions [80,81]. The Baader 220 claims to have the capability to perform portion process on small-sized fish, like Herring, Herb herring, and Sardine in 17–30 cm.

### 2.2. Metal Blade Design and Operation Parameters

Based on the type of machine and process parameters, various types of planar cutting blades are used in the filleting machine. The blades were produced from different types of carbon and alloy tool steels and high-grade stainless steel. For some important components, cryogenically hardened stainless steel with a polished surface could be utilized [82]. Handling fish products is crucial as these foods are susceptible to numerous degradation factors. Hence, the utensils, materials, and equipment used in this process must be sharp and easily maintained or available. Thus, stainless steel (corrosion resistant), aluminum, approved plastic material, and galvanized steel equipment are easily sanitized and cleaned [83]. A sharp knife or blade is essential in fish cutting as it improves the cutting moment, lowers grip force, and shortens the cutting time.

Researchers studied the shape effect of the cutting edge and the sharpness of knives on the cutting forces [84]. The following knife quality indicators are available for inspection under production conditions: sharpening angle, uniformity of width, straightness of the cutting edge, depth of chipping, absence of cracks on the cutting edge and sharpness of knives. The effect of the blade sharpening angle on the parameters of this process was analyzed [85]. They evaluated resistance force with different sharpness half-angle (5°, 10°, 20°, 50°) of the inclined back edge at different cutting speeds. With increasing the sharpening angle at low cutting speed (0–0.8 mm/s) and high cutting speed (10–25 mm/s), the resistance forces both decreased. Ageev et al. [15,86] also proposed theoretical concepts and determined the optimal parameters that influence the cutting process of the blade configurations. The fish samples were processed using a wire knife with different sharpness (0.025–0.400 mm), at different temperatures (2–12 °C), at different cutting speeds (2, 4, 33, 62, 91, and 126 mm/s) and with various blade thickness (1–5 mm) to evaluate the quantitative dependences of fracture resistance and friction resistance forces on each operation parameters. The force of fracture resistance rises nonlinearly as the knife’s sharpness and operational temperature increase. The force increases noticeably as the blade thickness increases. According to the measurement results, the total force of friction resistances reduces while fracture resistances increase as temperature and material elasticity increase. The reduction of undesirable energy consumption for cutting could be achieved by changing the geometry of the knife. This could be done by reducing the sharpening angle and the thickness of the knife, as well as by constructively introducing rear-inclined edges and eliminating the side edges [87]. The reduction of friction forces also could be facilitated by a decrease in the roughness of the edges, which was achieved by polishing and the use of anti-friction coatings [86,88]. Three different blades have been tested with different sliding angles and friction forces: bare blades (31.6°) and (35 µN), Ti-coated blades (20.3°) and (23.7 µN), and Z-TFMG (Zr-based thin film metallic glasses) coated blades (16.2°) and (19.2 µN). The results showed that the Teflon coating could reduce the cutting forces of an uncoated microtome blade by 80%, whereas the proposed Z-TFMG achieved a 51% reduction. Moreover, Z-TFMG was shown to protect blades during skin grafting surgery by providing a smooth surface morphology to reduce friction force and thereby improve blade sharpness. The finished fish product obtained by cutting must meet certain requirements for the accuracy of the shape, size, and smoothness of the cut. There is a compromise between cutting forces and flatness of the cutting surface that must be adopted in the design of the cutting tool. Besides the optimization of cutting conditions and geometry of the knives, the operation of cutting, and the fish product properties are also important factors contributing to reducing cutting forces and improving quality.

For a basic understanding of the cutting process as well as for modeling approaches, it is important to link operation conditions to undergoing cutting forces, including resistance and friction forces. Dowgiałło [89] proposed fundamental models for calculating the resistance forces that arise during fish cutting. The operating conditions are cutting with a flat knife with a thickness of b = 0.7 mm, which was sharpened on one side (β = 22°). And the length of the cutting line was a = 20 mm, and the speeds were applied according to the cut materials. The values of the cutting forces and pressure for sea and freshwater fish were determined for various regime parameters of processing with plate and circular knives. Ageev et al. [15,86] proposed a set of theoretical models for calculating contact pressures and resistance forces during fish cutting. A system of resistance forces that affect the knife during fish cutting was proposed, including the cutting speed (0.0–1.200 mm/s), at different rheological parameters (1.5–3.0 × 10^5^ N·s^−1^), the sharpness of the cutting tool (half-angles = 5°, 10°, 15°, 20°, 50°, 60°, 70°, 80° and 90°), the half-thickness of the knife (1.5, 2.0, 2.5, and 3.0 mm), and the elasticity of the fish muscle tissue (*e*= 3, 5, 7,11). The dimensional resistance force of the profile significantly depends on the rheological properties of the material. The sharpening angle and speed have very little influence on the resistance force that occurs during the cutting of the material; however, when cutting material in a low-viscous state, for example, upon defrosting and blanching, while with the increased blade thickness, the sharpening angle and speed have a noticeable influence on the value of the force in question. Jayraj et al. [90] also developed the relationship between friction and the inclination angles of fish on steel surfaces (20°–22°) and plastic surfaces (18°–20°) during feeding. By smoothing the interface between the fish’s body and the surface, the slime the fish secretes decreases the frictional impact. As a result, the surface of the belt conveyors used to transport fish for unit activities in fish processing should be rough or have grips. This should prevent the fish from moving. Furthermore, they also recorded the power consumption during fish slicing, which is related to the speed of the blade and fish weight. The freshly harvested fish samples are required to accurately measure the slicing force and power requirement because the textural parameters of skin hardness, stiffness, and toughness would decay, and the force required to slice the fish would be reduced [91]. The skin hardness ranged between 86.911 and 95.656 N within five days of storage and thereafter reduced within the range from 48.714 to 65.920 N. The stiffness ranged between 3.1474 and 4.6340 N·mm^−1^ and toughness, 588.9–713.2 N·mm for five days. After five days of storage, the stiffness and toughness reduced in the range of 2.0030–2.8111 N·mm^−1^ and 415.0–526.3 N·mm, respectively.

### 2.3. Drawbacks and Limitations

**Figure 3:**
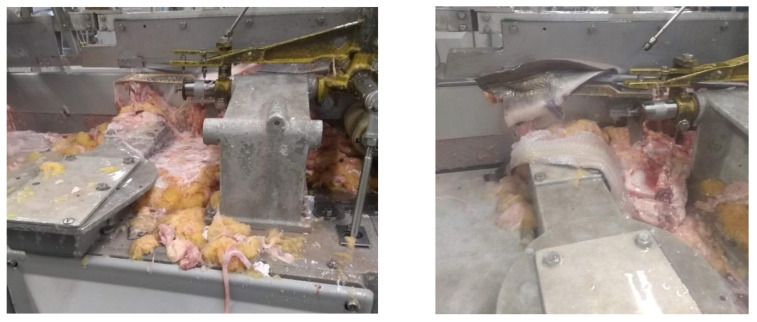
The filleting machine is frequently jammed because the fish guts are left in the machine. The normal filleting processing is affected by the jamming issue, which makes some fillets drop out of the machine as waste.

**Figure 4:**
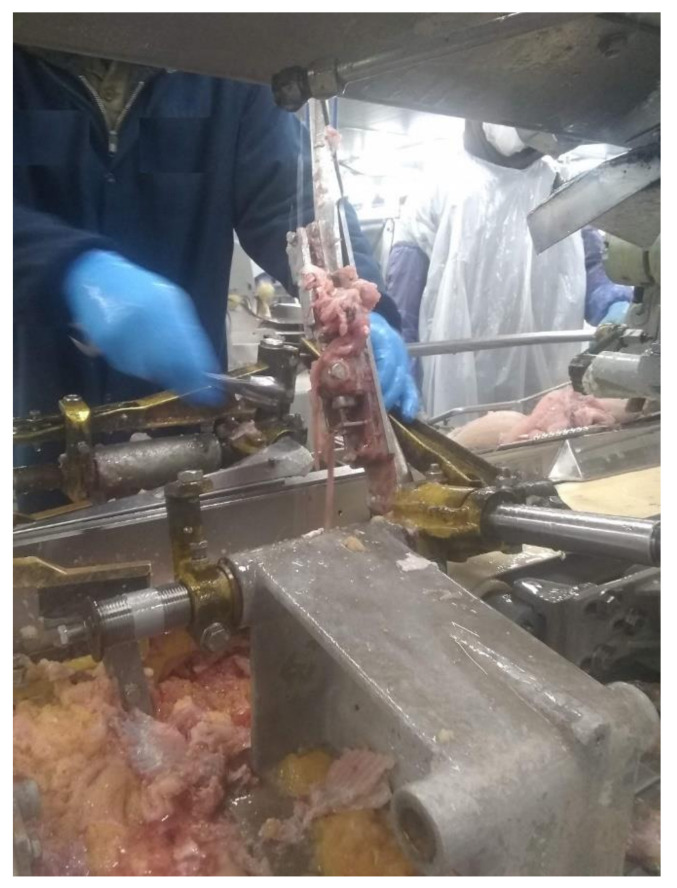
The manual cleaning process for the filleting machine.

**Figure 5:**
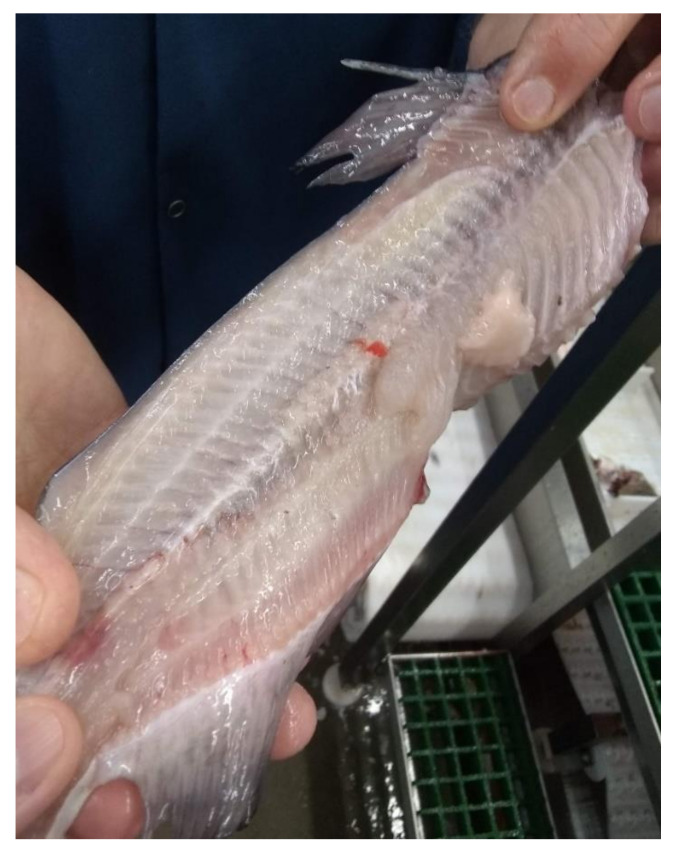
A fish rib bone from the filleting machine has a lot of fish meat left on it.

**Figure 6:**
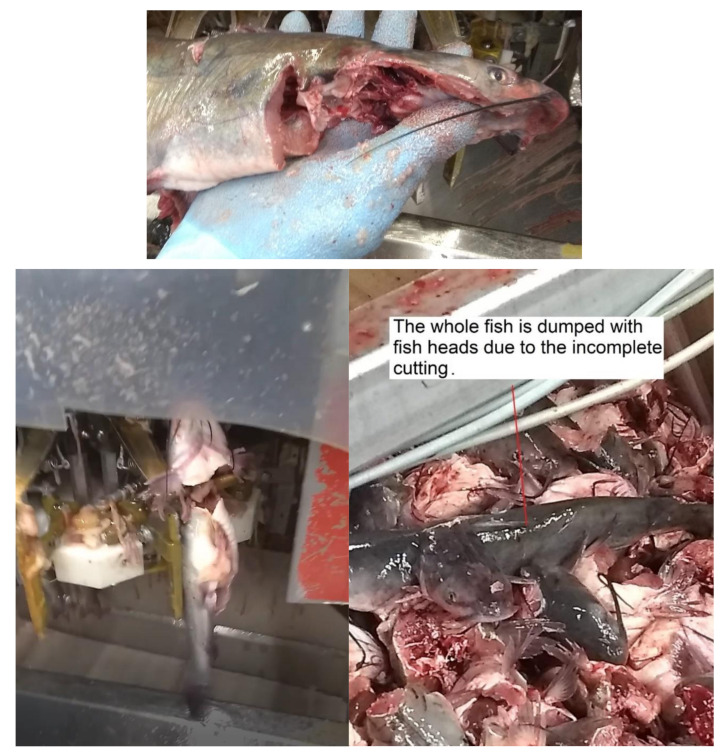
The waste due to deheading processes.

We have visited several fish-cutting industries to learn about the challenges and drawbacks of current fish-cutting technologies. Current metal blade cutting has too much manual and repetitive work in several cutting steps. Some fish-cutting facilities have no gutting step because of the cost and space limitations. Also, some machinery companies mention that their equipment eliminates the need for gutting the fish before filleting and saves at least one operation labor by combining header gutter and filleting machines. However, the cutting performances of the filleting machines can be easily affected by the jamming issues of the cutting blades since some fish guts, and other byproducts are left inside the machines, as shown in Figure 3. Therefore, a certain number of labors are required to keep monitoring the cleanliness of the cutting blades and do maintenance work (Figure 4). Figure 5 presents another issue with the metal blade cutting: low-level fish fillet yield and high-level waste. Even though some machines are designed to recycle the mid-rib bones for producing the mince [92], increasing the fish fillet yield can provide more income opportunities than post-processing the mid-rib bones. The metal blade cutting setup, such as the cutter gap, is designed for a specified range of fish sizes. Consequently, it will cause a lower yield for the smaller fishes, and smashing damage the bones of the larger fish. Especially the smashing damage of the bones, a small piece of the bones left inside the fish fillet may be stuck in people’s throats and lead to choking [93]. Fish larger than the size settings cannot be normally processed by machines, which will increase the waste. Another example of generating waste in the deheading machine is shown in Figure 6. The fish heads of the larger fish may not be fully cut off. The fish body is still partially connected with the fish head then the whole fish is dumped. Although some blades can be adjusted to fit different fish sizes, it is time-consuming and inefficient. Therefore, the current metal blade-based cutting technologies have an urgent need for innovation to increase fillet yield and reduce labor and turnover.

## 3. Waterjet Cutting

To revolutionize the catfish processing industry, it appears that a cost-effective non-conventional and non-contact cutting technology is needed with higher productivity, lower waste, and less labor-intensive requirement. The waterjet cutting technology can be an excellent alternative option to meet these expectations since it has many advantages over other traditional technologies. The excellent mobility and flexibility allow all directions cutting behaviors and complicated curved cutting lines [94]. Compared with laser cutting, there is no heat generated and no radiation zones, so lower cutting temperatures can guarantee the food’s freshness and extend shelf life [95]. Besides, cross-contamination and bacterial transmission can be prevented because of no blades [96]. In addition, a large quantity of water is required in traditional fish processing factories to clean the fish product for every processing step [97]. Therefore, the waterjet cutting process can realize the integration of the processing and cleaning functions. Furthermore, the jamming issue described above will not happen, so the cutting performance would be much more constant and steadier, and the maintenance and inspection labor can be saved. The modern waterjet cutting machines typically consist of: (1) a control unit including a micro-computer, cameras, and supporting software, (2) a waterjet table that can withstand the strong impact of water flow during cutting assays, (3) a position traverse system which is controlled by the micro-computer for adjusting the related position of the nozzle and target workpieces, (4) a high-pressure pump for generating enough high pressure for the water, (5) a waterjet nozzle with certain shape and diameters of die for passing the high-pressure waterjets [98]. High-pressure waterjet has been applied to several fish-cutting steps: portioning, trimming, and scaling [27,99,100,101]. Due to the development of automatic technologies in the past two decades, high-performance robot arms have been largely employed in recent industries [8,102]. With the integration of robots, advanced waterjet cutting can conduct a highly precise cutting process with high speed, which favors the efficiency of fish fillet yield production [102]. However, the current waterjet cutting techniques are still facing certain issues. Unlike cutting other materials, which require precise size control, factors such as relatively large kerf width, blind cuts, and taper-long cutting lines are not considered the top concerns in fish cutting. One of the major obstacles that block the application of waterjet in fish cutting is its relative slow cutting speed and much higher starting and maintenance costs compared to blade cutting due to the system’s complexity. In addition, waterjet cutting generates wastewater containing minced fish and bone. Disposal equipment and related specialized training will then furtherly increase the cost of waterjet fish cutting.

The design and development of waterjet cutting equipment for fish cutting aim to gain faster and cheaper processing, accurate workpieces identification, and automatic cutting strategy generation and operation. The performance of meat cutting can be evaluated in different grades with the fish fillet recovery rate, the damage of fish bones, the amount of saw mince, the cleanness of skin cut, the cleanness of muscle cut, and the cut-through of connective tissue [27,82,103]. To achieve the best cutting performance, the components of waterjet cutting should be well-designed. It is believed that factors majorly, including the water pressure and flow rate, transverse speed, nozzle shape and diameter, the nozzle stand-off distance, nozzle tilting angle, number of passes, and fish temperature, affect the overall waterjet cutting quality. The supplying water pressure is considered the most important parameter governing the waterjet cutting quality [104,105]. The water pressure varies according to the strength of the targeting materials and exhibits a different order magnitude [106]. By focusing on the rainbow trout, and fixing the stand-off distance and incidence angle, Kasperowicz et al. [32] have investigated the effect of the waterjet pressure, nozzle moving speed, nozzle size, and nozzle geometry on the cutting performance. It turns out that, for good cutting quality, the supply water pressure changes with the size of carcasses and cutting sites. For a full cut of the skinless lobe, the water pressure should reach 3.5 MPa or 35 atm. Within the selected nozzle size (0.175 mm–0.95 mm) and nozzle traverse speed (0–50 mm/s), no obvious effect on cutting quality was observed. The complexity of setting cutting parameters for specific fish meat is reflected in this study. This implies the fact that the fish waterjet cutting may also follow the principles summarized during the cutting assays of other workpieces. Research has also found that with a lower sample traverse speed, a smoother and deeper cutting cross-section was obtained [107]. This is important for obtaining high-quality cutting while increasing the raw fish meat yield. The quality of waterjet cutting is also influenced by the temperature of the fish meat [101]. The systematic study of cutting parameters for specific targeting materials in the food industry can be a hot topic for future research on waterjet cutting. Moreover, the fluid mechanical properties of the waterjet also affect the cutting performance and should be considered during the parameters design.

Although commercial waterjet cutting instruments have been applied to fish cutting to a certain extent, the fundamental research of waterjet cutting in different fish under different parameters still lacks and deserves more attention. The quantitative reports in the literature about the effect of these factors on fish cutting quality are very limited. One of the reasons is that the experimental data usually are trade secrets. On the other hand, it is difficult to obtain precise and universal experiment results because (1) the fish meat from the same species may have different properties according to the place and season [108]. (2) The structure and properties of fish meat may differ even within the same species [109]. Hence, reliable and universal results require extensive experiments. Nevertheless, one can still get insight into the effect of the design parameters on cutting performance by limiting the study variables.

## 4. Machine Vision and Artificial Intelligence for Fish Cutting

Fish cutting heavily relies on manual labor, and the working environment is usually cold and wet to ensure the freshness of the fish. In addition, many procedures involved in fish cutting are tedious, repetitive, and unsafe. Therefore, increasing automation levels of fish cutting have gained tremendous attention in recent years [110]. With the advancements in imaging and computing technologies, machine vision and AI have been applied to the fish industry for improved precision and automation levels of fish cutting.

Machine vision enables inspecting objects or scenes objectively and efficiently through image acquisition and analysis (e.g., object detection/localization, semantic/instance segmentation) to assist in automating various tasks (e.g., fish species sorting, cutting), which otherwise would have been done manually. Machine vision has been widely used for postharvest product inspection in food industries, including fish processing, such as fish morphology identification (e.g., size, volume, weight, and shape estimation) [111,112], species recognition [113,114], physical or chemical properties [8,18], and quality and damage inspection [115]. In general, machine vision relies on the acquisition, processing, and modeling of two-dimensional (2-D) and three-dimensional (3-D) images to assist in operations in fish processing.

There is considerable research on using 2D images in fish processing, where acquired color images are segmented for interested regions based on the fish/fillet color [22,116,117,118]. Several commercial fish cutting systems, such as Baader 988 [118] and Marel SensorX [119], used CCD cameras in the control systems of blades. For fish deheading machines (Section 2.1), the cutting position was decided based on the gill area of the fish. Accurate localization of fish gill regions would be hence critical in the cutter controller [13]. Jain et al. [22] used the machine vision system in a deheading machine. The authors fused the data from CCD cameras, optical encoders, and ultrasonic displacement to detect the collar bone position and adjust the blades accordingly. Such a machine vision system can potentially improve the accuracy of gill recognition and thus reduce protein waste during fish cutting. Sivertsen et al. [118] applied a CCD camera to scan cod fillets using the ridge detection algorithm, where the centerline of fillets can be identified with an average accuracy of 1 mm from the tail.

Since regular color cameras are limited to providing information in a 2D plane, 3D imaging systems are useful in fish processing lines by obtaining the depth information of target fish [120,121,122]. 3D imaging can be realized using techniques such as structured light, stereovision, or time-of-flight (ToF). The laser-scanning profilometry (line light-based 3D imaging), which only needs to capture the reflection of laser pulses and is not readily influenced by ambient light, can potentially achieve high measurement accuracy. This method can be employed in commercial fish-cutting systems, such as the Marel I-Cut series (Marel, 2022a), to empower a trimming robot with 6 degrees of freedom (DoF) for 3D cutting [122]. Bondø et al. [121] implemented a laser-based 3D vision system to obtain the point cloud data of fish with the accuracy of 1 mm, which was analyzed to detect the position of fish gill that can be used to guide a robot arm to cut along the gill arch.

In recent years, AI has been used to empower machine vision systems for enhanced fish cutting. AI through machine learning (ML), especially deep learning (DL), has demonstrated great remarkable performance in visual recognition tasks (e.g., object detection and segmentation). AI methods have been used in fish recognition to improve the blade-cutting accuracy of automated production lines [19,116,123,124,125]. They could be further implemented in fish deheading, trimming, and portioning operations. Gamage et al. [19] conducted pioneering work to apply ML in fish-cutting tasks. Fish head images were acquired with a CCD camera and processed to obtain edge-enhanced, 2D Gaussian-smoothed images. Features were then computed from enhanced images and fed into multiple regression algorithms to estimate the point of interest on the fish head. Odone et al. [126] proposed a machine vision-based fish grading system. This system performed fish shape measurement and used support vector machine (SVM) models to learn the relation between fish weight and shape parameters, enabling the fish grading at a rate of 3 items per second.

DL methods have been recently used in machine vision systems for fish processing. Xu and Sun [127] used convolutional neural networks (CNNs) to detect the salmon muscle gaps to reduce irregular voids or undesirable lace-like appearance in the final product. Taheri-Garavand et al. [128] constructed a CNN classifier to classify a caught fish into fresh and non-fresh with an overall accuracy of 98.21%. Laradji et al. [129] proposed a CNN-based point-level fish segmentation model achieving an accuracy of 87.9%. Diamond et al. [37] used U-Net to perform the segmentation of different parts of the fish on 2D images. The dataset of fish images used in the study was made open-source.

These studies demonstrate the promise of machine vision and AI as valuable tools for automated fish processing. Their full potential in fish cutting, however, remains to be investigated, given only a handful of relevant publications. A key question remains to be answered as to how satisfactorily machine vision and AI-driven fish cutting automated machines will perform in commercial production settings. Although more validation experiments, especially with real-time machine vision prototyping, are urgently needed, it is generally agreed that assistance from machine vision and AI will substantially automate fish-cutting operations and reduce labor costs.

Further, there is still a great deal of machine vision and AI that is worth investigating and can be potentially applied in fish cutting and whole fish industries. Studies [130,131] have shown that AI methods can be used for the optimization of cutting parameters of fish cutting machines. For cutting fault diagnosis, the precise location of fault points can be obtained by supervised pattern classifiers [132,133,134]. Moreover, vision- and speech-based multimodal human-machine interaction systems can be applied to fish-cutting machines to provide workers with a better operating experience [135]. Machine vision and AI algorithms can power waterjet cutting or metal blade cutting for improved fish cutting productivity. For instance, Lin et al. [136] proposed a machine vision and image recognition guided waterjet knife strawberry berry calyx removal machine. Similar approaches based on machine vision and AI can be applied to finer fish cutting operations. In addition, AI technology can also be used to create a reliable and intelligent metal blade wear detection system to mitigate the negative effects of the fish-cutting processes [137].

## 5. Future Perspectives

Major manufacturers of fish-cutting devices around the world have been summarized in Table 1. Potential directions for future research on fish-cutting techniques are presented as follows:

(1) Some fish species deserve more attention, such as catfish. Catfish was ranked 8th in the top 10 seafood of U.S. per capita consumption in 2019 [138]. Mississippi and Alabama were ranked first and second in catfish farming and production in 2021, respectively [139]. However, compared with 300 million kilograms in 2003, the U.S. catfish industry’s yield decreased to 136.5 million kilograms in 2014 because of international market competition and increasing feed costs [140]. Furthermore, compared with other fish species, catfish have just a few rib bones, so the catfish belly flap portion can be trimmed and sold as fish nuggets [141]. Therefore, developing special cutting machines or trimming and portioning methods for catfish can keep catfish farming and fillet processing industries (with a total impact of more than one billion dollars) as an important agricultural and food industry, which their existence is vital to the success of the US rural economy.
(2) Waterjet cutting should be further developed for other fish-cutting steps. Although the waterjet cutting works well for fish fillet portioning and trimming steps, there is no waterjet cutting application for other fish cutting steps, such as filleting and deheading. Also, the small-scale fish cutting processes are all performed by a metal blade system, and no waterjet cutting machine is designed or modified for small-scale fish. The cutting parameters can be adjusted to reach a critical condition: the water stream will cut along the edge of the fish meat and touch the fish bones but cut the meat only, which means that the water cutting force is adjusted to be just good for cutting meat but not strong enough for cutting bones. Under critical conditions, waterjet cutting can realize the maximum fish fillet yield. Besides, there is no research on abrasive waterjet fish cutting. It can be a better solution for further improving the fish-cutting quality and efficiency in deheading and filleting processing steps. The abrasives, including salt, sugar, ice, and starch particles, can be added to the high-pressure waterjet, which can reduce the surface roughness, favor energy saving, and lower the overall costs of equipment maintenance [33,34,142,143].
(3) Other novel cutting techniques should be developed for the fish-cutting process. Another two common food cutting techniques, ultrasonic cutting and laser cutting, are currently not good for fish processing. Even though some companies have designed ultrasonic cutting for slicing frozen fish [144], regular raw fresh fish is too soft, which cannot promise cutting quality and efficiency. Laser cutting will have heat-affected zones, which reduces the shelf life and quality of the fish meat. As potential directions for future research, ultrasonic cutting and laser cutting techniques can be adapted into fish cutting. Other novel direct-contact and non-contact cutting techniques can also be developed for fish-cutting innovations.
(4) Fully automated cutting production lines and integrated control systems should be designed by advancing singulation, machine vision, and AI technologies. Current automation cutting machines need manual singulation and placement processes for fish supply. Novel singulation methods can help realize fully automated systems to further reduce the total cutting and processing time, labor, and turnover. Considering the uniqueness and characteristic of each fish, the machine vision system in fish cutting lines could still be improved in both hardware and software aspects. As for the hardware system, the machine vision system should be able to capture the depth information. Therefore, the 3D sensing system, such as a laser profilometer or stereo cameras, could provide more comprehensive detail to guide the fish-cutting process. Meanwhile, due to the high computation requirement of 3D information, the data processing efficiency should be improved to satisfy the high-speed requirement of fish cutting lines. As for software development, first, key-point detection is widely used in machine vision applications and has made great progress in the past few years, where it can be applied to detect critical points in fish [145], such as gill [13] and mouth [146,147]. If the key points on the fish can be correctly located, this can facilitate the machine to cut the fish more accurately. Second, training DL models with large parameters is another major trend in machine vision and AI for visual pattern recognition tasks. Many studies have demonstrated that DL models better learn the representations of images. Therefore, visual transformer [148] and self-supervised learning [149,150] are two other techniques that can be used to extract meaning information from fish images. A better image feature extractor helps the system to analyze fish images and optimize fish-cutting operations.
(5) More research and simulation development should be devoted to quantifying and predicting fish cutting quality and efficiency. Especially for waterjet cutting, the scientific reports only define the cutting quality and efficiency for metals, woods, composite materials, and other food species. Except for the cutting grade definition, there is no specified parameter quantifying fish cutting quality and efficiency. Some parameters, such as surface roughness, need to be further investigated and adapted to evaluate the fish-cutting quality. As for simulation development, current simulation models for cutting processes and quality are mainly based on regression analysis, response surface methodology (RSM), computational fluid dynamics (CFD), and finite element method (FEM) [151,152,153,154]. Since the waterjet cutting method has great potential for fish cutting, future fish cutting simulation and theory development should pay more attention to pure and abrasive waterjet cutting processes of whole fish and fish fillets with higher accuracy and robustness.

## 6. Conclusions

The fisheries and fish farming industries today operate in a large and global market. In such an increasingly competitive marketplace, it becomes necessary for the fisheries and fish farming industries to explore advanced technological solutions for improving productivity and profitability. As the key part of the fish processing industry, efficient cutting of fish is important given that top quality, maximum yield, and highest possible profits are to be pursued keenly. This paper evaluates and summarizes the current fish cutting techniques and highlights the research on cutting mechanisms, blade design, and performance effect factors. The applications of machine vision and AI are reviewed and discussed. With the proposed future perspectives, it could be possible to bridge the knowledge gap on cutting operations and achieve better cutting efficiency and quality.

## Figures and Tables

**Table 1:** Major manufacturers of fish-cutting devices around the world.

Manufacturers	Cutting Operations	Fish Species	Product Advantages	Country	Reference
Arenco	Deheading, Filleting, Skinning	Pelagics	Stainless steel fish pockets carry the fish to a precision measuring device that customizes the position of the head cut and tail cut for each individual fish, achieving a high yield; The skinning machine works with around 300 fish per minute.	Sweden	[59,60]
BAADER	Deheading, Filleting, Portioning, Skinning, Trimming	Salmonids, Tuna, White fish, Pelagics, Aquaculture	Committing to 100% fish utilization and zero waste; Sophisticated automated cutting systems for quality fish products; Smart inspection systems and software provide meaningful insights and detailed reports for improved production control.	Germany	[50,53,54,55,69,70]
Cabinplant	Skinning	Pelagics	Thermal and mechanical treatments for skinning; Only 5% product loss; Limited amount of wastewater and no disposal of hazardous fluids.	Denmark	[7]
Cretel	Skinning	White fish, Pelagics, Aquaculture	Easy thickness control for silver, regular and deep skin.	Belgium	[71]
JBT	Portioning, Trimming	Salmonids, Tuna, White fish, Aquaculture	Computerized scanning of every piece coming into the machine, and sophisticated programming that controls waterjet portioners, horizontal slicers, 3D portioners, X-ray guided solutions, and systems with multiple cutting heads for portioning, slicing, stripping, and dicing.	USA	[73,122]
Kaj Olesen	Portioning, Skinning	Salmonids, White fish, Aquaculture	The slicing machine has a capacity of up to 250 slices per minute; The thickness of the slices can be adjusted from 1 mm up to 5 mm, and can slice fillets from the smallest to the largest sizes; Without the use of any tools, the salmon slicer can be transformed into a tuna slicer—only by changing the “tower” of the machine.	Denmark	[10]
Kroma	Deheading, Filleting	Salmonids, Pelagics, Aquaculture	The fish is turned during head cutting to obtain the U cut; The workplaces for the operators are designed with maximum consideration for ergonomics.	Denmark	[146,147]
Marel	Deheading, Filleting, Portioning, Skinning, Trimming	Salmonids, Tuna, White fish, Aquaculture	Filleting and trimming lines streamline processing with less product handling and continuous data collection. The fish is weighed, graded, cut, and packed in a continuous flow, increasing capacity, throughput and labor efficiency; Have both metal blade cutting and waterjet cutting technologies to create an endless variety of value-added products, from fixed-sized strips, dices, and splits to high-value portions of fixed weight and length.	Iceland	[27,51,80,101]
MARELEC Food Technologies	Portioning, Trimming	Salmonids, White fish	Have both metal blade cutting and waterjet cutting technologies. Max Waterjet pressure 600 MPa; High accuracy with machine vision technologies; Flexible cutting algorithms for portioning.	Belgium	[73,78,79]
Nikko	Deheading, Filleting, Portioning	Salmonids, White fish, Pelagics, Aquaculture	The filleting machine can do both center cutting and regular filleting without having to re-arrange the line; The “Kamaless Header” cuts the fish head with the collar intact to increase yield.	Japan	[9]
NOCK Maschinenbau	Skinning	Salmonids, White fish, Aquaculture	CBF and SB machine models are suitable for thin, pulling-off the skin, and deep skinning conditions.	Germany	[64,65]
PERUZA	Deheading, Filleting	Pelagics	Laser fish measurement system to realize the maximum yield.	Latvia	[46,61]
Pisces Fish Machinery	Deheading, Filleting	Salmonids, White fish, Pelagics, Aquaculture	A complete range of filleting systems can process fish from 10 g to 10 kg; Incorporating automatic positioning of each individual fish to ensure the head cut is in the optimum position for maximum yield; Some machines allow for head-on gutted fish to be processed into fillets with one operator.	USA	[47,48]
ROSOMA	Deheading, Portioning	Salmonids, White fish, Pelagics, Aquaculture	On the basis of a fish typical of deheading, the head cutting angle desired is set up by adjusting the tappets among one another; For pelagic fish, the machine can do beheading and subsequent gutting by means of a vacuum. A suction extractor consisting of a vacuum pump and a separator forms part of the vacuum nobbing.	Germany	[25]
Ryco Equipment	Deheading, Filleting, Portioning	Salmonids, White fish, Aquaculture	Collar-on head cutting with maximum recovery to process fish from 1 to 50 pounds.	USA	[56,57,58]
Salmco	Portioning	Salmonids	The product range offers everything from simple hand slicers to semi- and fully automatic cold slicers and various fresh slicers with a variable 0° to 90° cutting angle.	Germany	[81]
SEAC AB	Deheading, Filleting	Pelagics	Machines can process fish down to 70–100 pieces/kg at speeds up to 320 fish/min; The accuracy of processing is up to 98%.	Sweden	[46]
Skaginn 3X	Skinning	White fish, Pelagics, Aquaculture	Skinning after SUB-CHILLING™ preserves the texture of the fillets for better quality and texture and lower yield loss.	Iceland	[70]
STEEN	Deheading, Skinning, Trimming	Salmonids, Tuna, White fish, Pelagics, Aquaculture	Skinning and de-frilling in two perfectly synchronized units.	Belgium	[82]
Tecnotrans UG	Portioning, Skinning	Salmonids, Tuna, White fish, Aquaculture	Both fresh and frozen goods can be processed to cut weight-accurate and uniform slices & cubes.	Germany	[6]
TOYO SUISAN KIKAI	Deheading, Filleting, Portioning, Skinning, Trimming	Salmonids, Tuna, White fish, Pelagics	Cover all the cutting steps and a wide range of fish species.	Japan	[11,92]
Uni-Food Technic	Deheading, Filleting, Portioning, Skinning	Salmonids, White fish, Pelagics	Produce intelligent automatic fish machinery that reduces the need for labor costs; Complete filleting lines with de-heading machines, de-sliming machines, de-scaling machines, trimming lines with yield control, skinning machines, and pin-bone removers.	Denmark	N/A
VARLET	Portioning, Skinning	Salmonids, White fish	Slicing offers a constant thickness adjustment from 5 to 10mm with a dismountable spiked pusher; Skinning has a single or double track with a removable conveyor.	France	N/A
Velfag	Deheading, Filleting, Skinning	White fish	Deheading is designed to both head whole fish and take the collarbone off pre-headed fish; Filleting has the capability of handling large white fish (20 kg+); The combined skinning machines attached directly to the filleting machines allow the fillets to be skinned directly, with no extra employees needed between filleting and skinning.	Iceland	[115]

## Data Availability

The data presented in this study are available on request from the corresponding author.

## References

[1] Bjørndal T., Brasão A., Ramos J., Tusvik A. (2016). Fish processing in Portugal: An industry in expansion. Mar. Policy.

[2] Jayathilakan K., Sultana K., Radhakrishna K., Bawa A.S. (2012). Utilization of byproducts and waste materials from meat, poultry and fish processing industries: A review. J. Food Sci. Technol..

[3] Jeebhay M.F., Robins T.G., Lopata A.L. (2004). World at work: Fish processing workers. Occup. Environ. Med..

[4] FAO (2022). The State of World Fisheries and Aquaculture 2022.

[5] Adeyeye S.A.O. (2016). Traditional fish processing in Nigeria: A critical review. Nutr. Food Sci..

[6] Aldás Guerrero R.F. (2013). Diseño de un Negocio Dedicado a la Exportación de Filete de Tilapia en Camas Frías al Mercado Canadiense Período 2014–2018. Bachelor’s Thesis.

[7] Buljo J., Gjerstad T. (2013). Robotics and automation in seafood processing. Robotics and Automation in the Food Industry.

[8] Einarsdóttir H., Guðmundsson B., Ómarsson V. (2022). Automation in the fish industry. Anim. Front..

[9] Nagaishi H., Inada T., Yoshioka T., Sato A. (2017). Development of a compact, onboard slurry icemaker to rapidly produce optimal ice for maintaining freshness of marine products. Synth. Engl. Ed..

[10] Thordarson G., Karlsdottir M., Pedersen R., Johannsson M., Hognason A. (2015). Sub-Chilling of Salmon.

[11] Tsukagoshi T., Uchita T. Toyo Suisan Kikai Co Ltd, 2019. Method for Removing Guts of Fish Body and Device for Same. U.S. Patent.

[12] Shirai K., Ramirez-Ramirez J.C. (2010). Utilization of Fish Processing By-products for Bioactive Compounds. Fish Processing: Sustainability and New Opportunities.

[13] de Silva C., Wickramarachchi N. (1997). An innovative machine for automated cutting of fish. IEEE/ASME Trans. Mechatron..

[14] Lang H., Wang Y., de Silva C.W. (2008). An automated industrial fish cutting machine: Control, fault diagnosis and remote monitoring. Proceedings of the 2008 IEEE International Conference on Automation and Logistics.

[15] Ageev O.V., Dowgiałło A., Sterczyńska M., Piepiórka-Stepuk J., Giurgiulescu L., Janowicz M., Jakubowski M. (2021). Experimental characterization and theoretical modeling of fracture and friction resistance forces during tuna cutting. J. Food Eng..

[16] Ashwinkumar N., Bhuvaneshkumar S., Adithya K. (2021). Development and Study of Universal Fish Cutting Apparatus. Int. J. Res. Eng. Sci. Manag..

[17] Kamaruzzaman K.A., Mahfurdz A., Hashim M., Bidin M.N. (2020). Design and Performance Evaluation of Semi-Automatic Fish Cutting Machine for Industry. IOP Conference Series: Materials Science and Engineering.

[18] Dowlati M., de la Guardia M., Mohtasebi S.S. (2012). Application of machine-vision techniques to fish-quality assessment. TrAC Trends Anal. Chem..

[19] Gamage L.B., De Silva C.W., Gosine R.G. (1993). Statistical pattern recognition for cutter positioning in automated fish processing. Proceedings of the IEEE Pacific Rim Conference on Communications Computers and Signal Processing.

[20] Arvanitoyannis I.S., Kassaveti A. (2008). Fish industry waste: Treatments, environmental impacts, current and potential uses. Int. J. Food Sci. Technol..

[21] Goossens Y., Schmidt T.G., Kuntscher M. (2020). Evaluation of Food Waste Prevention Measures—The Use of Fish Products in the Food Service Sector. Sustainability.

[22] Jain A., De Silva C.W., Wu Q.M.J. (2001). Intelligent fusion of sensor data for product quality assessment in a fish cutting machine. Proceedings of the Joint 9th IFSA World Congress and 20th NAFIPS International Conference (Cat. No. 01TH8569).

[23] Atkins A., Xu X. (2005). Slicing of soft flexible solids with industrial applications. Int. J. Mech. Sci..

[24] Liu S., Wang H., Cai Y. (2021). Research on Fish Slicing Method Based on Simulated Annealing Algorithm. Appl. Sci..

[25] A Saltykov M., I Tkachenko T. (2021). Multidimensional Classification for Systematization of Fish Processing Equipment. IOP Conf. Series Earth Environ. Sci..

[26] Tomaszewska-Gras J. (2016). Rapid quantitative determination of butter adulteration with palm oil using the DSC technique. Food Control.

[27] Franklínsdóttir H. (2014). Application of Water Jet Cutting in Processing of Cod and Salmon Fillets. Master’s Thesis.

[28] Schreuders F.K.G., Sagis L.M.C., Bodnár I., Boom R.M., van der Goot A.J. (2022). Non-linear rheology reveals the importance of elasticity in meat and meat analogues. Sci. Rep..

[29] Bogue R. (2008). Cutting robots: A review of technologies and applications. Ind. Robot. Int. J. Robot. Res. Appl..

[30] Khodabandehloo K. (2022). Achieving robotic meat cutting. Anim. Front..

[31] Carreño-Olejua R., Hofacker W.C., Hensel O. (2010). High-Pressure Water-Jet Technology as a Method of Improving the Quality of Post-Harvest Processing. Food Bioprocess Technol..

[32] Kasperowicz M.B., Chomka G.P., Bil T. (2019). Determination of Supply Pressure during Cutting Fish Using High-Pressure Water Stream Taking into Account the Cutting Place and Diameter of the Water Nozzle. Int. J. Food Eng..

[33] McGeough J. (2016). Cutting of Food Products by Ice-particles in a Water-jet. Procedia CIRP.

[34] Wang J., Shanmugam D. (2009). Cutting meat with bone using an ultrahigh pressure abrasive waterjet. Meat Sci..

[35] Pogrebnyak A., Pogrebnyak V. (1567). Mechanism of the High Efficiency of the Cutting Frozen Food Products Using Water-Jet with Polymer Additions. Food Sci. Technol..

[36] Hao M., Yu H., Li D. (2015). The measurement of fish size by machine vision—A review. Proceedings of the International Conference on Computer and Computing Technologies in Agriculture.

[37] Diamond K.M., Avants B.B., Maga A.M. (2021). Machine learning-based segmentation and landmarking of 2D fish images. Integrative and Comparative Biology.

[38] Xu W., Wang J., Deng Y., Li J., Yan T., Zhao S., Yang X., Xu E., Wang W., Liu D. (2022). Advanced cutting techniques for solid food: Mechanisms, applications, modeling approaches, and future perspectives. Compr. Rev. Food Sci. Food Saf..

[39] Bland J.M., Bett-Garber K.L., Li C.H., Brashear S.S., Lea J.M., Bechtel P.J. (2018). Comparison of sensory and instrumental methods for the analysis of texture of cooked individually quick frozen and fresh-frozen catfish fillets. Food Sci. Nutr..

[40] Buckingham R., Graham A., Arnarson H., Snaeland P., Davey P. (2001). Robotics for de-heading fish—A case study. Ind. Robot. Int. J..

[41] Ketels D. (2008). Apparatus for Positioning Fish for Heading. U.S. Patent.

[42] Sampels S. (2015). The effects of processing technologies and preparation on the final quality of fish products. Trends Food Sci. Technol..

[43] Dowgiallo A. (2008). The effect of cutting and fish-orientation systems on the deheading yield of carp. Int. J. Food Sci. Technol..

[44] Tomczak-Wandzel R., Vik E.A., Wandzel T. (2015). BAT in Fish Processing Industry: Nordic Perspective.

[45] Martín Rodríguez F., Barral Martínez M. (2015). Automatic turbot fish cutting using machine vision. Instrum. Viewp..

[46] Sharapov S. (2013). Compact Design of Fish Processing Equipment and Implementation of Lean Tools. Master’s Thesis.

[47] Kaufman D., Fisher R.A., Wanchese Fish Company Feasibility Study for Machine Processing Croakers into Fillets and for Forming the Fillets into Larger Portions. Fishery Resource Grant FRG 1999 - 24. Virginia Institute of Marine Science, William & Mary. https://scholarworks.wm.edu/reports/2216.

[48] Wastell T.T. (2021). Pisces Fish Machinery Inc. Fish Filleting Machine. U.S. Patent.

[49] Rora A.M.B., Mørkøre T., Einen O., Kestin S.C., Warriss P.D. (2001). Primary processing (evisceration and filleting). Farmed Fish Quality.

[50] Braeger H., Scherch R.P. (2001). Baader North America Corp. Process for Fileting Fish and Machine for Performing This Process. U.S. Patent.

[51] Jacobsen P.H., Jakobsen B.K. (2014). Marel Salmon, A.S. Fish filleting machine. U.S. Patent.

[52] Jakobsen B., Jacobsen P.H. (2006). Carnitech, A.S. Fish Filleting Machine. U.S. Patent.

[53] Kowalski W. (2015). Nordischer Maschinenbau Rud Baader GmbH; Co, K.G. Method for Removing Blood Released during Filleting from the Backbone of Fish, and Device for Removing Such Blood. U.S. Patent.

[54] Kowalski W. (2016). Nordischer Maschinenbau Rud Baader GmbH; Co, K.G. Method for Mechanically Removing Pin Bones from Fillet Parts of Conveyed Fish and Device for Performing Said Method. U.S. Patent.

[55] Jürs M., Schroeder M. (2014). Nordischer Maschinenbau Rud Baader GmbH; Co, K.G. Apparatus and Method for Filleting Beheaded and Eviscerated Fish. U.S. Patent.

[56] Ryan R.M. (2013). RYCO EQUIPMENT Inc. Fish Processing System and Method. U.S. Patent.

[57] Ryan R.M. (2014). RYCO EQUIPMENT Inc. Fish Processing System and Method. U.S. Patent.

[58] Ryan R.M. (2017). RYCO EQUIPMENT Inc. Fish Processing Systems and Methods. U.S. Patent.

[59] Sone I., Sveinsdóttir H.I., Stefánsson G., Larsson K., Undeland I., Skåra T., Romotowska P.E., Karlsdóttir M.G. (2020). Investigating commercially relevant packaging solutions to improve storage stability of mechanically filleted Atlantic mackerel (*Scomber scombrus*) produced under industrial conditions. Eur. Food Res. Technol..

[60] Sveinsdóttir H.I., Karlsdóttir M.G., Arason S., Stefánsson G., Sone I., Skåra T., Rustad T., Larsson K., Undeland I., Gudjónsdóttir M. (2020). Effect of antioxidants on the sensory quality and physicochemical stability of Atlantic mackerel (*Scomber scombrus*) fillets during frozen storage. Food Chem..

[61] Da Mota A.M. (2019). Optimização da Estratégia de Serviço Pós-Venda da Peruza. Ph.D. Thesis.

[62] Thrane M., Nielsen E.H., Christensen P. (2009). Cleaner production in Danish fish processing–experiences, status and possible future strategies. J. Clean. Prod..

[63] Zieliński B., Kapłonek W., Nadolny K. (2018). Regeneration of industrial cutting blades made from X39Cr13 steel used in skinning process of Pleuronectidae-family flatfishes. J. Mech. Energy Eng..

[64] Schwarz O. (2015). Nordischer Maschinenbau Rud Baader GmbH; Co, K.G. Conveying Apparatus Comprising a Conveying Path and Designed to Supply a Plurality of Products for Consumption Having Soft Parts to a Processing Device, and Processing Machine Comprising a Conveying Apparatus and a Processing Device. U.S. Patent.

[65] Schwarz O. (2018). Nordischer Maschinenbau Rud Baader GmbH; Co, K.G. Device and Method for Removing a Surface Layer Including the Skin from Fish Fillets. U.S. Patent.

[66] Arnesen J.A., Gildberg A. (2007). Extraction and characterisation of gelatine from Atlantic salmon (Salmo salar) skin. Bioresour. Technol..

[67] Bland J.M., Grimm C.C., Bechtel P.J., Deb U., Dey M.M. (2021). Proximate Composition and Nutritional Attributes of Ready-to-Cook Catfish Products. Foods.

[68] Zieliński B., Kapłonek W., Sutowska M., Nadolny K. (2019). Analysis of a Feasibility Study of a Precision Grinding Process for Industrial Blades Used in the Cutting of Soft Tissues by a Prototype 5-Axis CNC Grinding Machine. Appl. Sci..

[69] Joensen S., Olsen J.V. (2003). Bløt Hyse. Spalting av Hysefilet Etter Skinning.

[70] Arnþórsdóttir M.G., Arason S., Margeirsson B. (2008). Combined Blast and Contact.

[71] Waterston S.W., Holmes J.D. (2005). The Fish-Skinning Machine: An Unusual Source Of Hand Trauma. Plast. Reconstr. Surg..

[72] Ørnholt-Johansson G., Gudjónsdóttir M., Nielsen M.E., Skytte J.L., Frosch S. (2017). Analysis of the production of salmon fillet—Prediction of production yield. J. Food Eng..

[73] Mathiassen J.R., Misimi E., Bondø M., Veliyulin E., Østvik S.O. (2011). Trends in application of imaging technologies to inspection of fish and fish products. Trends Food Sci. Technol..

[74] Grasselli G. (2014). Industrial Slicer. U.S. Patent.

[75] Grasselli G. (2017). Industrial Slicer. U.S. Patent.

[76] Ross K., Edwards J. (2015). Spatial Variation in the Mercury Concentration of Muscle Myomeres in Steaks of Farmed Southern Bluefin Tuna. Foods.

[77] Singh A., Surasani V.K.R. (2020). Fish processing: An entrepreneurial opportunity for livelihood and income generation. J. Krishi Vigyan.

[78] Lorentzen G., Ageeva T.N., Heia K. (2021). Desalting of dried salt-cured cod (*Gadus morhua* L.) without water renewal-3D imaging of volume change. Food Control.

[79] Sture Ø., Øye E.R., Skavhaug A., Mathiassen J.R. (2016). A 3D machine vision system for quality grading of Atlantic salmon. Comput. Electron. Agric..

[80] Bro T. (2015). Marel Salmon, A.S. D-Cut Slicer. U.S. Patent.

[81] Manchay Aparco L.D. (2020). Evaluación de Conservas en Base a Productos Hidrobiológicos. Bachelor’s Thesis.

[82] Kapłonek W., Nadolny K., Zieliński B., Plichta J., Pimenov D.Y., Sharma S. (2020). The Role of Observation–Measurement Methods in the Surface Characterization of X39Cr13 Stainless-Steel Cutting Blades Used in the Fish Processing Industry. Materials.

[83] Faostat: Statistical Databases. http://faostat.fao.org/.

[84] Karltun J., Vogel K., Bergstrand M., Eklund J. (2016). Maintaining knife sharpness in industrial meat cutting: A matter of knife or meat cutter ability. Appl. Ergon..

[85] Viatcheslavovich A.O., Arkadievich N.V., Adgamovich F.Y. (2019). Mathematical simulation of knife profile resistance force during fish cutting. Вестник Астраханскoгo Гoсударственнoгo Техническoгo Университета. Серия: Рыбнoе Хoзяйствo.

[86] Ageev O.V., Fatykhov Y., Ivanova E.E. (2020). Optimization of the knife profile for resource-saving primary fish processing. News of institutes of higher education. Food Technol..

[87] Ageev O., Naumov V.A., Fatykhov J.A. (2019). Mathematical Modeling of the Resistance Force of the Profile of a Flat-Back Knife. J. Frict. Wear.

[88] Chu J.P., Diyatmika W., Tseng Y.-J., Liu Y.-K., Liao W.-C., Chang S.-H., Chen M.-J., Lee J.-W., Jang J.S.C. (2019). Coating Cutting Blades with Thin-Film Metallic Glass to Enhance Sharpness. Sci. Rep..

[89] Dowgiallo A. (2005). Cutting force of fibrous materials. J. Food Eng..

[90] Jayraj P., Machavaram R., Sahu G., Paradkar V. (2019). Measurement of Morphometric Dimensions and Mechanical Properties of Rohu Fish for Design of Processing Machines. J. Aquat. Food Prod. Technol..

[91] Jain D., Pathare P.B., Manikantan M. (2007). Evaluation of texture parameters of Rohu fish (Labeo rohita) during iced storage. J. Food Eng..

[92] Yamase S., Tsukagoshi T., Morita K., Takeuchi K., Obara T., Maloney P.J. (2011). Toyo Suisan Kikai Co Ltd; Nippon Suisan, K.K.; UniSea Inc. Method of Separation of Backbone Part of Fish and Device Therefor. U.S. Patent.

[93] Vallamkondu V., Carlile S., Shakeel M., Ah-See K.W. (2013). Neck abscess and vocal cord paresis: Delayed complications of a self-extruded long fishbone stuck in throat. BMJ Case Rep..

[94] Liu X., Liang Z., Wen G., Yuan X. (2019). Waterjet machining and research developments: A review. Int. J. Adv. Manuf. Technol..

[95] Krajcarz D. (2014). Comparison Metal Water Jet Cutting with Laser and Plasma Cutting. Procedia Eng..

[96] Wulfkuehler S., Stark S., Dietz J., Schmidt H., Weiss A., Carle R. (2014). Effect of Water Jet Cutting and Moderate Heat Treatment on Quality of Fresh-Cut Red Oak Leaf Lettuce (*Lactuca sativa* L. var. crispa). Food Bioprocess Technol..

[97] Muthukumaran S., Baskaran K. (2013). Organic and nutrient reduction in a fish processing facility—A case study. Int. Biodeterior. Biodegrad..

[98] Hace A., Jezernik K. (2004). Control system for the waterjet cutting Machine. IEEE/ASME Trans. Mechatron..

[99] Huang S.-W., Chou J.-H., Tsai J.-T. (2018). Uniform Design and Regression Analysis Methods for Optimal Operational Parameter Design of High-pressure Waterjet Machine. Int. J. Autom. Smart Technol..

[100] Omar F.K., de Silva C.W. (2000). Optimal portion control of natural objects with application in automated cannery processing of fish. J. Food Eng..

[101] Thorarinsdottir K.A. (2015). APRICOT-Automated Pinbone Removal in Cod and Whitefish.

[102] Barbut S. (2020). Meat industry 4.0: A distant future?. Anim. Front..

[103] Barbut S. (2014). Review: Automation and meat quality-global challenges. Meat Sci..

[104] Folkes J. (2009). Waterjet—An innovative tool for manufacturing. J. Mater. Process. Technol..

[105] Irwansyah I., Ibrahim M., Ferdiansyah H. (2012). Influence of water-jet nozzle geometry on cutting ability of soft material. J. Rekayasa Kim. Lingkung..

[106] Kasperowicz M., Chudy J., Chomka G. (2018). Determining the supply pressure depending on the feed speed and the diameter of the nozzle. Carpathian J. Food Sci. Technol..

[107] Pogrebnyak A., Pogrebnyak V., Perkun I., Vasyliv N. (2020). Influence of geometric and dynamic parameters of a water-polymer jet on characteristics of food products hydro-cutting process. Ukr. Food J..

[108] Sandor Z., Papp Z.G., Csengeri I., Jeney Z. (2011). Fish meat quality and safety. Sci. J. Meat Technol..

[109] Hyldig G., Nielsen D. (2001). A review of sensory and instrumental methods used to evaluate the texture of fish muscle. J. Texture Stud..

[110] Komlatsky V.I., Podoinitsyna T.A., Verkhoturov V.V., Kozub Y.A. (2019). Automation technologies for fish processing and production of fish products. J. Phys. Conf. Ser..

[111] Kong F., Tang J., Rasco B., Crapo C., Smiley S. (2007). Quality Changes of Salmon (Oncorhynchus gorbuscha) Muscle during Thermal Processing. J. Food Sci..

[112] Mohd R.M.S., Amjad R., Rosely K., Norhaida A., Tanzila S. (2012). FiLeDI framework for measuring fish length from digital images. Int. J. Phys. Sci..

[113] Sharmin I., Islam N.F., Jahan I., Joye T.A., Rahman R., Habib T. (2019). Machine vision based local fish recognition. SN Appl. Sci..

[114] Storbeck F., Daan B. (2001). Fish species recognition using computer vision and a neural network. Fish. Res..

[115] Tveit G.M., Sistiaga M.B., Øye E.R., Schei M. (2017). Kvalitetsvurdering av Fisk Fanget Med to-og Fire-Panels Seleksjonsinnretninger: Bidrar 4-Panelkonstruksjoner og Knuteløst lin til Økt Kvalitet? Tokt Ombord F/Tr Havtind 28.06. 16–11.07. 16.

[116] Azarmdel H., Mohtasebi S.S., Jafari A., Muñoz A.R. (2019). Developing an orientation and cutting point determination algorithm for a trout fish processing system using machine vision. Comput. Electron. Agric..

[117] Misimi E., Erikson U., Skavhaug A. (2008). Quality Grading of Atlantic Salmon (Salmo salar) by Computer Vision. J. Food Sci..

[118] Sivertsen A.H., Chu C.-K., Wang L.-C., Godtliebsen F., Heia K., Nilsen H. (2009). Ridge detection with application to automatic fish fillet inspection. J. Food Eng..

[119] Andersen K. (2009). Processing Quality Seafood. International Seafood Trade: Challenges and Opportunities.

[120] Bar E., Mathiassen J.R., Eilertsen A., Mugaas T., Misimi E., Linnerud S., Salomonsen C., Westavik H. (2016). Towards robotic post-trimming of salmon fillets. Ind. Robot. Int. J. Robot. Res. Appl..

[121] Bondø M.S., Mathiassen J.R., Vebenstad P.A., Misimi E., Bar E.M.S., Toldnes B., Østvik S.O. (2011). An automated salmonid slaughter line using machine vision. Ind. Robot. Int. J. Robot. Res. Appl..

[122] Mathiassen J.R., Misimi E., Østvik S.O., Aursand I.G. (2012). Computer vision in the fish industry. Computer Vision Technology in the Food and Beverage Industries.

[123] Sun M., Yang X., Xie Y. (2020). Deep learning in aquaculture: A review. J. Comput..

[124] Xu J.-L., Sun D.-W. (2017). Identification of freezer burn on frozen salmon surface using hyperspectral imaging and computer vision combined with machine learning algorithm. Int. J. Refrig..

[125] Zhao S., Zhang S., Liu J., Wang H., Zhu J., Li D., Zhao R. (2021). Application of machine learning in intelligent fish aquaculture: A review. Aquaculture.

[126] Odone F., Trucco E., Verri A. (2001). A trainable system for grading fish from images. Appl. Artif. Intell..

[127] Xu J., Sun D.-W. (2017). Computer Vision Detection of Salmon Muscle Gaping Using Convolutional Neural Network Features. Food Anal. Methods.

[128] Taheri-Garavand A., Nasiri A., Banan A., Zhang Y.-D. (2020). Smart deep learning-based approach for non-destructive freshness diagnosis of common carp fish. J. Food Eng..

[129] Laradji I., Saleh A., Rodriguez P., Nowrouzezahrai D., Azghadi M.R., Vazquez D. (2020). Affinity lcfcn: Learning to segment fish with weak supervision. arXiv.

[130] Savkovic B., Kovac P., Rodic D., Strbac B., Klancnik S. (2020). Comparison of artificial neural network, fuzzy logic and genetic algorithm for cutting temperature and surface roughness prediction during the face milling process. Adv. Prod. Eng. Manag..

[131] Tanikić D. (2020). Computationally intelligent optimization of metal cutting regimes. Measurement.

[132] Choudhary A., Mian T., Fatima S. (2021). Convolutional neural network based bearing fault diagnosis of rotating machine using thermal images. Measurement.

[133] He M., He D. (2017). Deep Learning Based Approach for Bearing Fault Diagnosis. IEEE Trans. Ind. Appl..

[134] Jia F., Lei Y., Guo L., Lin J., Xing S. (2018). A neural network constructed by deep learning technique and its application to intelligent fault diagnosis of machines. Neurocomputing.

[135] Abioye A.O., Prior S.D., Thomas G.T., Saddington P., Ramchurn S.D. (2018). The multimodal speech and visual gesture (mSVG) control model for a practical patrol, search, and rescue aerobot. Annual Conference Towards Autonomous Robotic Systems.

[136] Lin J., Holmes M., Vinson R., Ge C., Pogoda F.C., Mahon L., Gentry R., Seibel G.E., Chen X., Tao Y. (2017). Design and testing of an automated high-throughput computer vision guided waterjet knife strawberry calyx removal machine. J. Food Eng..

[137] Marani M., Zeinali M., Kouam J., Songmene V., Mechefske C.K. (2020). Prediction of cutting tool wear during a turning process using artificial intelligence techniques. Int. J. Adv. Manuf. Technol..

[138] (2019). National Marine Fisheries Service (2021) Fisheries of the United States, 2019. U.S. Department of Commerce, NOAA Current Fishery Statistics No. https://www.fisheries.noaa.gov/national/sustainable-fisheries/fisheries-united-states.

[139] USDA United States Department of Agriculture (2022). Catfish Production Reports from National Agricultural Statistics Service (NASS).

[140] Tan Y., Gao H., Chang S.K., Bechtel P.J., Mahmoud B.S. (2018). Comparative studies on the yield and characteristics of myofibrillar proteins from catfish heads and frames extracted by two methods for making surimi-like protein gel products. Food Chem..

[141] Hill J.I., Nelson R.G., Woods K.L., Weese J.O., Whitis G.N. (2013). Consumer preferences for attributes of catfish nuggets: Price, breading color, cooking method, and country of origin. Aquac. Econ. Manag..

[142] Ashrafi N. Viscoelastic abrasive waterjet. Proceedings of the ASME International Mechanical Engineering Congress and Exposition.

[143] Shakouri E., Abbasi M. (2018). Investigation of cutting quality and surface roughness in abrasive water jet machining of bone. Proc. Inst. Mech. Eng. Part H J. Eng. Med..

[144] Sonikel Ultrasonics Frozen Fish Slicing with Ultrasonic. https://www.youtube.com/watch?v=nAe071BoMFY.

[145] Wang N., Gao X., Tao D., Yang H., Li X. (2018). Facial feature point detection: A comprehensive survey. Neurocomputing.

[146] Kristensen I., Jorgensen D.B., Kroma A.S. (2016). Fish Processing Machine and a Method for Processing Fish. U.S. Patent.

[147] Kristensen I., Jorgensen D.B., Kroma A.S. (2017). Fish Processing Machine and a Method Enabling That Fish Can Be Processed through the Mouth. U.S. Patent.

[148] Dosovitskiy A., Beyer L., Kolesnikov A., Weissenborn D., Zhai X., Unterthiner T., Dehghani M., Minderer M., Heigold G., Gelly S. An image is worth 16x16 words: Transformers for image recognition at scale. arXiv.

[149] Chen T., Kornblith S., Norouzi M., Hinton G. A simple framework for contrastive learning of visual representations. In International Conference on Machine Learning. Proceedings of the International Conference on Machine Learning.

[150] He K., Fan H., Wu Y., Xie S., Girshick R. (2020). Momentum contrast for unsupervised visual representation learning. Proceedings of the IEEE/CVF Conference on Computer Vision and Pattern Recognition.

[151] Annoni M., Arleo F., Malmassari C. (2014). CFD aided design and experimental validation of an innovative Air Assisted Pure Water Jet cutting system. J. Mater. Process. Technol..

[152] Gzaiel M., Triki E., Barkaoui A. (2019). Finite element modeling of the puncture-cutting response of soft material by a pointed blade. Mech. Mater..

[153] Hu H., Li H., Wang Q., He J., Lu C., Wang Y., Liu P. (2020). Anti-blocking performance of ultrahigh-pressure waterjet assisted furrow opener for no-till seeder. Int. J. Agric. Biol. Eng..

[154] Polyakov A., Zhabin A., Averin E., Polyakov A. (2019). Generalized equation for calculating rock cutting efficiency by pulsed water jets. J. Rock Mech. Geotech. Eng..

